# Are all species necessary to reveal ecologically important patterns?

**DOI:** 10.1002/ece3.1246

**Published:** 2014-12-02

**Authors:** Edwin Pos, Juan Ernesto Guevara Andino, Daniel Sabatier, Jean-François Molino, Nigel Pitman, Hugo Mogollón, David Neill, Carlos Cerón, Gonzalo Rivas, Anthony Di Fiore, Raquel Thomas, Milton Tirado, Kenneth R Young, Ophelia Wang, Rodrigo Sierra, Roosevelt García-Villacorta, Roderick Zagt, Walter Palacios, Milton Aulestia, Hans ter Steege

**Affiliations:** 1 Ecology and Biodiversity Group, Utrecht UniversityUtrecht, the Netherlands; 2 Section Botany, Naturalis Biodiversity CenterLeiden, the Netherlands; 3 Department of Integrative Biology, University of CaliforniaBerkeley, California, 94720-3140; 4 IRD, UMR AMAPMontpellier, France; 5 The Field Museum1400 S. Lake Shore Drive, Chicago, Illinois, 60605-2496; 6 Center for Tropical Conservation, Nicholas School of the Environment, Duke UniversityDurham, North Carolina, 27708; 7 Endangered Species Coalition8530 Geren Rd., Silver Spring, Maryland, 20901; 8 Universidad Estatal AmazónicaPuyo, Ecuador; 9 Universidad Central Herbario Alfredo Paredes, Escuela de Biología Herbario Alfredo ParedesAp. Postal 17.01.2177, Quito, Ecuador; 10 Wildlife Ecology and Conservation & Quantitative Spatial Ecology, University of Florida110 Newins-Ziegler Hall, PO Box 110430, Gainesville, Florida; 11 Department of Anthropology, University of Texas at AustinSAC 5.150, 2201 Speedway Stop C3200 Austin, Texas, 78712; 12 Iwokrama International Programme for Rainforest ConservationGeorgetown, Guyana; 13 GeoISEl Día 369 y El Telégrafo, 3° Piso, Quito, Ecuador; 14 Geography and the Environment, University of TexasAustin, Texas, 78712; 15 Northern Arizona UniversityFlagstaff, Arizona, 86011; 16 Institute of Molecular Plant Sciences, University of EdinburghMayfield Rd, Edinburgh, EH3 5LR, UK; 17 Royal Botanic Garden of Edinburgh20a Inverleith Row, Edinburgh, EH3 5LR, UK; 18 Tropenbos InternationalLawickse Allee 11, PO Box 232, Wageningen, 6700 AE, the Netherlands; 19 Universidad Técnica del Norte, Herbario Nacional del EuadorQuito, Ecuador; 20 Herbario Nacional del EcuadorCasilla 17-21-1787, Avenida Río Coca E6-115, Quito, Ecuador

**Keywords:** Beta-diversity, Fisher's alpha, indets, large-scale ecological patterns, Mantel test, morpho-species, nonmetric multidimensional scaling, similarity of species composition, spatial turnover

## Abstract

While studying ecological patterns at large scales, ecologists are often unable to identify all collections, forcing them to either omit these unidentified records entirely, without knowing the effect of this, or pursue very costly and time-consuming efforts for identifying them. These “indets” may be of critical importance, but as yet, their impact on the reliability of ecological analyses is poorly known. We investigated the consequence of omitting the unidentified records and provide an explanation for the results. We used three large-scale independent datasets, (Guyana/ Suriname, French Guiana, Ecuador) each consisting of records having been identified to a valid species name (identified morpho-species – IMS) and a number of unidentified records (unidentified morpho-species – UMS). A subset was created for each dataset containing only the IMS, which was compared with the complete dataset containing all morpho-species (AMS: = IMS + UMS) for the following analyses: species diversity (Fisher's alpha), similarity of species composition, Mantel test and ordination (NMDS). In addition, we also simulated an even larger number of unidentified records for all three datasets and analyzed the agreement between similarities again with these simulated datasets. For all analyses, results were extremely similar when using the complete datasets or the truncated subsets. IMS predicted ≥91% of the variation in AMS in all tests/analyses. Even when simulating a larger fraction of UMS, IMS predicted the results for AMS rather well. Using only IMS also out-performed using higher taxon data (genus-level identification) for similarity analyses. Finding a high congruence for all analyses when using IMS rather than AMS suggests that patterns of similarity and composition are very robust. In other words, having a large number of unidentified species in a dataset may not affect our conclusions as much as is often thought.

## Introduction

In comparative ecology, the proper naming of species is essential. Historically, ecological studies have assigned a particular name to a particular entity based on the Darwinian species concept, which uses morphological characters to separate clusters of individuals into species (Darwin 1859; Mallet 2008). While studying ecological patterns at large scales, ecologists are often unable to identify all individuals encountered in the field to species. This leads to a potential problem: individuals that are recorded in a dataset but which have no valid species name (hereafter “indets”). As databases grow larger, so does the number of indets, with each plot added to a database also adding a number of new unidentified morpho-species (UMS), which ecologists must either incorporate or ignore in analyses. Both of these options potentially introduce errors of some sort, and there is no agreement among ecologists how indets should be handled or to what degree they might compromise the results of large-scale analyses.

These questions have been addressed on multiple occasions. Pitman et al. (1999), comparing tree species communities, also raised the question what would be the result of eliminating species that lacked taxonomic identification. In their view, the only variable that would substantially change with more individuals identified to a species was the geographic range of a species (Pitman et al. 1999). Following this statement, Ruokolainen et al. (2002) focused on the geographical ranges of the identified versus unidentified species previously mentioned by Pitman et al. (1999), agreed that this bias has the potential to greatly distort analyses, and added that it is not necessarily confined to distributional patterns. Some might be more obvious than others; species richness will be underestimated when unidentified specimens belong to new species, and this will also affect the relative abundance distribution. Similarities of species composition may also be affected, which will affect subsequent analyses that depend on these similarities, importantly Mantel tests and ordinations, tests that are often used by ecologists.

Many studies have sought a middle ground between high-cost, taxonomically precise analyses and more cost-effective methods without losing valuable ecological information, for instance, by relaxing taxonomic resolution (Terlizzi et al. 2003; and references therein) or by randomly reassigning UMS to identified species present in other plots or to itself again, in which case it was considered a new species (Cayuela et al. 2011). This, however, unintentionally increases similarity between plots. In several studies, correlations were in fact found between different taxon-level approaches and the patterns in abundance and composition in both marine and terrestrial habitats (Vanderklift et al. 1998; Pik et al. 1999, 2002; Enquist et al. 2002). In an attempt to abbreviate forest inventories, Higgins & Ruokolainen also made use of higher-taxon-level analyses by eliminating species identifications (Higgins and Ruokolainen 2004). While promising, these studies mostly dealt with unidentified species by decreasing taxonomic resolution, allowing the use of more individuals from a dataset without identification up to species level. However, as Terlizzi et al. (2003) have noted, many large-scale ecological questions (e.g., species loss or the degradation of forest diversity) require species-level analyses.

While new analytical tools offer some help in standardizing ecological datasets, removing synonyms, and checking the validity of names (e.g., the Taxonomic Name Resolution Service (TNRS: Boyle et al. 2013) and the R packages *taxize* and *taxonstand*), they cannot help solve the indet problem. In a theoretical approach, it was shown that, by subsampling datasets at random, thereby simulating a random sampling at a lower intensity, and by making subsamples based on the difficulty in identifying them, the outcome of analyses on species richness and composition does not necessarily change (Vellend et al. 2008). The first probably being the result of the relative abundance distribution theoretically remaining identical even with smaller subsamples, because of the random sampling. To our knowledge, the effect of omitting unidentified species has not yet been tested with actual data containing unidentified records at a scale as presented here.

Here, we use three independent large-scale harmonized and standardized tree inventory datasets (Guyana/Suriname, French Guiana and Ecuador) to test whether ecological patterns such as species diversity, richness, composition, and underlying gradients in the full datasets, using all morpho-species differ from those in subsets of identified morpho-species. This was done using three often-used analyses: species richness and Fisher's alpha (Fisher et al. 1943), to study patterns in tree species diversity, the similarity of species composition between samples for studying patterns in species turnover (Nekola and White 1999) and nonmetric multidimensional scaling (NMDS), an ordination technique designed to search for patterns in community composition. We also tested the similarities using a higher taxon level, in this case, genus-level, against results generated by the complete dataset (i.e., all morpho-species, the sum of the identified morpho-species and unidentified morpho-species included). These tests have significant practical implications, because a finding of no difference between using only identified morpho-species or all morpho-species would suggest a simple solution to the indet problem: omitting them altogether. In turn, this might make it possible to use large datasets that are currently underutilized in ecology because they contain large numbers of indets.

## Methods

### Species composition data

**Figure 1:**
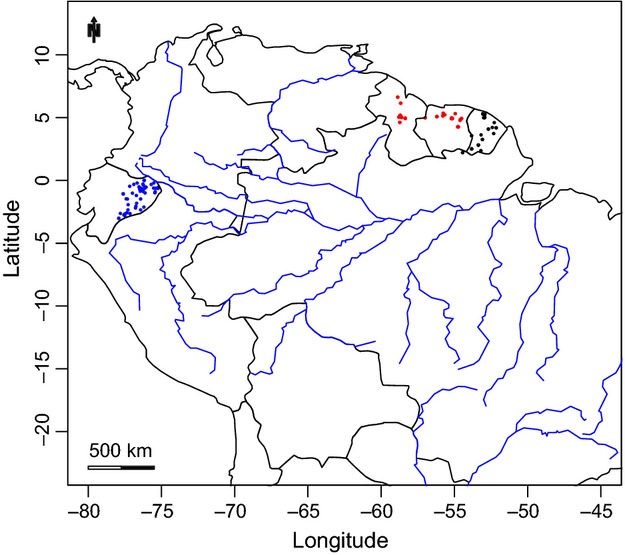
Map showing location of all 202 plots belonging to the Ecuador (blue), Guyana/Suriname (red), and French Guiana (black) datasets.

Three independent, nonoverlapping, tree inventory datasets were assembled: one from Guyana and Suriname, one from Ecuador, and one from French Guiana (Fig. 1). Each dataset consisted of 63–72 one-hectare plots, in which all trees ≥10 cm DBH had been inventoried (see Table 1 for details). Within each dataset, one or two persons responsible for the majority of the collected material harmonized all species names. Olaf Bánki and Juan Ernesto Guevara performed harmonization for the Guyana/Suriname and Ecuador datasets, respectively, while Daniel Sabatier and Jean-François Molino together harmonized the French Guianan dataset (hereafter referred to as OSB, JEG, S-M). Harmonization was done by morphological comparison of collections with reference to a “morpho-holotype” for each morpho-species. Species names of all subsets were standardized with the W3 Tropicos database, using TNRS (Boyle et al. 2013). The three datasets were harmonized independently of each other; no attempt was made to harmonize the three datasets into one.

**Table 1:** The number of one-hectare plots for each forest type listed by country. Guyana and Suriname are used as one dataset. Type abbreviations are Igapó (IG), Podzol (PZ), Swamp (SW), Terra Firme (TF), and Várzea (VA). Minimum diameter at breast height (DBH) as limit for measurement was 10 centimeters for all plots

	IG	PZ	SW	TF	VA	Min. DBH	Nr. 1-Ha plots
Guyana/Suriname	0	21	0	45	1	10	67
Ecuador	2	3	4	53	10	10	72
French Guiana	0	0	0	63	0	10	63
Total	2	24	4	161	11	NA	202

Three types of common ecological analyses (described below) were performed for each dataset twice: once for the all morpho-species (hereafter AMS) and once for a subset composed of only identified morpho-species (IMS), omitting the unidentified morpho-species of this dataset (UMS – thus AMS = IMS + UMS). All tests were performed in the “R” statistical and programming environment (R Core Team 2012). To calculate the Mantel statistics and metaMDS (a variant of NMDS), we used the package “*vegan”* (Oksanen et al. 2013). All linear models were tested for significance with a permutation procedure from the package “*lmperm”* (Wheeler 2010).

### Diversity analyses

To test how UMS influence analyses of alpha- and beta-diversity, we calculated Fisher's alpha values (Fisher et al. 1943) for every one-hectare plot twice: once with AMS and once for only IMS. We then performed a linear regression analysis between Fisher's alpha calculated for AMS and IMS to determine whether diversity patterns remain the same when datasets are truncated like this. Fisher's alpha is a widely used diversity index, specifically suited for species abundances following a logseries distribution. Fisher's alpha has been shown to be a very efficient diversity index for discriminating between sites (Taylor et al. 1976). This is a consequence of Fisher's alpha being theoretically independent of sample size, and therefore, much less influenced by the abundances of the more common species (Kempton 1979; Condit et al. 1998). If UMS can safely be excluded from the dataset, we expect to find no deviation from the pattern predicted by using only IMS or AMS and high *R*^2^ values from the linear regression analysis. We do expect, however, as UMS are especially common among the rare species, that omitting UMS may result in a significant decrease in Fisher's alpha, which was tested by a paired sample t-test.

### Similarity in species composition

To examine whether floristic similarity between plots differed when using AMS or only IMS, we constructed floristic similarity matrices for each dataset and a geographical distance matrix between the plots. Again, this was done twice for each dataset: once for AMS and once for IMS. We calculated the Mantel statistic (Mantel 1967) as the matrix correlation between the two similarity matrices (in this case, the floristic and the geographical matrix). Random permutation of both rows and columns of the species similarity matrix is then used to evaluate the significance of the performed test (Legendre and Fortin 2010). We performed a linear regression between the pairwise similarities between all plots of each dataset to assess the prediction of similarity values based on only the IMS. Because the two similarity matrices (i.e., based on IMS only or AMS) are not independent, this should be interpreted as underestimates of the risk to abandon the null hypothesis of nondependence between the matrices. However, we need to stress that despite the nonindependence, this is exactly the test we need to perform as we are interested whether IMS are a good predictor of AMS. Floristic similarity values were first calculated with the Bray–Curtis index of similarity, which is based on both species occurrence and abundances at each site (Bray and Curtis 1957). For comparison, we also used the Jaccard index and the Sørensen index to calculate similarities. The Jaccard index is only based on species presence or absence, ignoring differences in species abundance (Jaccard, 1901) and calculates similarity as the number of shared species between two sites divided by the total number of species of the two sites combined. The Sørensen index (Sørensen, 1948) is in essence much the same as the Jaccard index with the exception of giving double the weight to the shared species. To test the degree to which pairwise communities are more different or more similar than expected by chance, we used the Raup–Crick distance metric and repeated the above analyses. The Raup–Crick metric (*β*_RC_) was previously used in paleontological studies and just recently in some works related to variation in beta-diversity and species turnover (Raup and Crick 1979; Anderson et al. 2011; Chase et al. 2011; Kraft et al. 2011). The *β*_RC_ metric calculates the similarity between two communities under a null model. Assuming that SS_1,2_ is the number of shared species between two communities with values of alpha-diversity *α*_1_ and *α*_2_, respectively, the *β*_RC_ is obtained by random draws of *α*_1_ and *α*_2_ species from a determined species pool to estimate the probability of observing the shared species. The Mantel statistic was first calculated based on the standard distance matrix function in Vegan *Vegdist*. We then used the Raup–Crick method, under a null model assuming that the occurrence probability of species is frequency dependent, and performed the Mantel's statistic and linear regression on the matrices of pairwise similarities again. Similar to the diversity analyses, if omitting UMS from our datasets indeed makes no difference, we again expect to find high *R*^2^ values from the regression between analyses performed on IMS and AMS. In addition, we also tested for the deviation from a slope of 1 belonging to the relationship of *y* = *x* (i.e., when IMS and AMS generate the exact same results). To test whether using a higher taxon approach would yield similar results as the approach based on AMS as above, we also tested results from a similarity analysis based on only genera against the results of the AMS dataset. Agreement between similarities was analyzed using the same procedure as above.

### Multivariate analyses

To evaluate the underlying structures of floristic composition within the three datasets, we performed nonmetric multidimensional scaling (NMDS) using MetaDMS. Two NMDS were performed separately for each dataset: one for AMS and one for IMS. The scores of the first and second axes were then compared separately by linear regression. NMDS is an ordination technique, which attempts to find the best rank-order agreement between actual similarities in floristic similarity and interpoint distance in the computed ordination space (Fasham 1977; Minchin 1987; Salako et al. 2013). NMDS therefore does not try to fit axes based on eigenvalues, but instead represents a coordinate system for the ordination space. We used metaMDS, a NMDS procedure that centers the origin on the averages of the axes and uses principal components to align the scores in such a way that most variation is projected along the first axis (Oksanen et al. 2013). We tested the hypothesis that the patterns produced by the NMDS on the first and second axes are similar using either the IMS or AMS and hence that linear regressions will yield high *R*^2^ values. Here, we also tested for the deviation from a slope of 1 belonging to the relationship of *y* = *x*.

### Data stratification

To test for the robustness of predictions based on IMS, we created random smaller subsets to perform the same Mantel test as explained above. A random subset of, respectively, 50% and 25% was selected from the Guyana/Suriname, French Guiana, and Ecuador IMS pool. In making the IMS dataset even smaller in comparison with the complete dataset (by randomly omitting IMS), we simulated a larger proportion of UMS. This was repeated for 50 iterations from which mean values were calculated for the similarity matrices using the same three indices as used for the similarity analyses described above.

## Results

### Floristic composition and level of species identification

The proportion of IMS varied in the three datasets from 44–77%. In Guyana and Suriname (OSB), 67 plots yielded 37,446 individual trees, for a total of 1042 AMS and 458 IMS (44%). The mean number of UMS per plot was 27 with a median of 24. Mean fraction of IMS per plot for Guyana/Suriname was 70%. Ecuador (JEG) with a total of 72 plots yielded 34,544 individual trees, for a total of 2021 AMS and 1391 IMS (69%), with a mean number of 17 and a median of 16 UMS per plot. The mean proportion of IMS for each plot in Ecuador was 90%. In French Guiana (S-M), 63 plots yielded 35,075 individuals of trees, for a total of 1204 AMS and 925 IMS (77%). Mean number of UMS per plot was 15 with a median of 15. The mean proportion of IMS per plot in French Guiana was 91%. Linear regressions between the number of AMS and the number of IMS were high, with *R*^2^ values of 0.938, 0.976, and 0.959 for Guyana/Suriname, Ecuador, and French Guiana, respectively.

### Predicted species diversity based on identified morpho-species

Linear regressions between Fisher's alpha (FA) calculated using AMS and only the IMS were extremely high, yielding *R*^2^ values of >0.95 for all three datasets (Table 2). The slope of the linear model based on the Guyana/Suriname was 1.6. Using a 95% confidence interval for the slope showed that this was significantly different from the relation *y* = *x* with slope 1 (i.e., when there is no difference between FA based on AMS or just IMS). This was the case for Ecuador and French Guiana as well, with slopes of 1.12 and 1.10, respectively. As expected, FA showed an increase with an increasing number of species per plot for both IMS and AMS. FA calculated for just IMS ranged between 2.87–44.92 for Guyana/Suriname, 8.96–114.65 for Ecuador, and 27.61–114.65 for French Guiana. When using AMS, this was (in the same order) 4.65–78.17, 12.23–130.32, and 27.61–130.32. These differences were found to be significant after performing a paired sample t-test with significance levels for rejecting the *H*_0_ of equal ranges with probabilities <0.005 for all three datasets.

**Table 2:** Overview of all adjusted *R*^2^ coefficients from the linear regression for each analysis; listed for all three datasets. All regression coefficients were found significant at a 0.001 significance level after 5000 permutation iterations. Results of the stratification were averaged over 50 runs for each diversity index

	Guyana/Suriname	Ecuador	French Guiana
Valid versus Morpho			
Fisher's Alpha	0.967	0.959	0.970
Mantell Bray–Curtis	0.983	0.998	0.999
Mantell Bray–Curtis (genus level)	0.739	0.805	0.904
Mantell Jaccard	0.983	0.998	0.999
Mantell Sørensen	0.966	0.995	0.996
Raup–Crick	0.918	0.955	0.967
NMDS axis 1	0.979	0.998	0.9997
NMDS axis 2	0.991	0.988	0.998
Stratification (50%) Bray–Curtis	0.80 (SD 0.17)	0.92 (SD 0.042)	0.92 (SD 0.05)
Stratification (50%) Sørensen	0.60 (SD 0.073)	0.85 (SD 0.02)	0.81 (SD 0.051)
Stratification (50%) Jaccard	0.78 (SD 0.19)	0.91 (SD 0.04)	0.92 (SD 0.05)
Stratification (25%) Bray–Curtis	0.59 (SD 0.2)	0.81 (SD 0.07)	0.82 (SD 0.09)
Stratification (25%) Sørensen	0.51 (SD 0.12)	0.75 (SD 0.06)	0.71 (SD 0.097)
Stratification (25%) Jaccard	0.59 (SD 0.19)	0.79 (SD 0.072)	0.81 (SD 0.095)

### Patterns in morpho-species abundance

Because the slope between FA calculated for only the IMS and AMS deviated significantly from 1, we examined the rank abundance curves for both IMS and AMS for each dataset. The AMS datasets were consistently richer in species, especially the rare ones, when compared to the IMS datasets (Fig. 3). Moving from the AMS dataset to the IMS, more species were lost than individuals, significantly affecting FA. For instance, the IMS dataset contains only approximately 21% of the number of singletons compared to the AMS dataset in Guyana/Suriname. For Ecuador and French Guiana, this was 41% and 55%, respectively. In terms of numbers, there are a total of only 44 singletons in the IMS dataset of Guyana/Suriname against 210 in the AMS dataset (Ecuador = 212 vs. 518 and French Guiana = 114 vs. 208).

### Similarity in species composition

**Figure 2:**
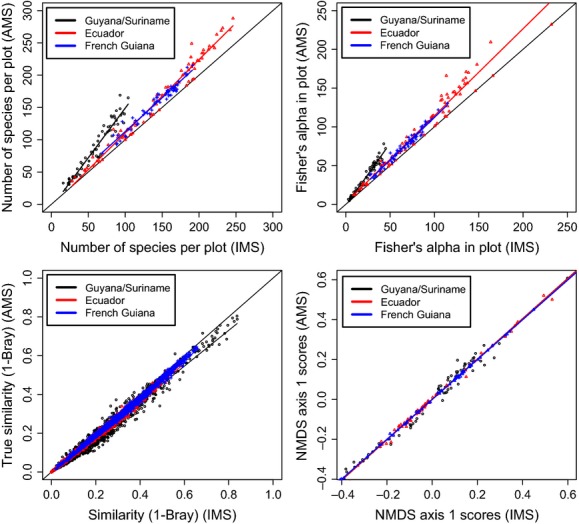
Comparisons between the IMS and AMS dataset for species richness per plot (top left), Fisher's alpha (top right), pairwise similarities between all plot pair combinations (bottom left), and axis 1 scores of the nonmetric multidimensional scaling (bottom right). All analyses were performed on the three large subsets Guyana/Suriname (o), Ecuador (Δ), and French Guiana (+). All analyses show extremely similar results and yield high *R*^2^ values.

Using IMS only, the similarity in species composition based on Bray–Curtis was predicted very well for all three datasets (*R*^2^ values of >0.98) (Table 2), and the slope in all cases was almost identical to 1 (Fig. 2). Confidence intervals showed, however, that, despite high adjusted *R*^2^ values, slopes from the linear regressions actually deviated significantly from 1 for all datasets when using the Bray–Curtis index (Guyana/Suriname CI 0.917–0.927, Ecuador 0.958–0.961, and French Guiana 0.979–0.982). The difference between using the Jaccard, Bray–Curtis, or Sørensen index for calculating similarities among plots appeared to be negligible, all resulted in adjusted *R*^2^ values of >0.96 (Table 2) with slopes from the linear regressions all still significantly deviating from 1 (for Jaccard: Guyana/Suriname CI 0.897–0.907, Ecuador 0.950–0.953, and French Guiana 0.973–0.976 and for Sørensen Guyana/Suriname CI 0.915–0.930, Ecuador 0.932–0.938, and French Guiana 0.969–0.974). Adjusted *R*^2^-values using the Raup–Crick distance metric yielded values of >0.91 for all three datasets. Examples of the patterns of distance decay with AMS, and only IMS can be found for all three datasets in the Supplementary Online Material (SOM). The Mantel's r coefficient for Guyana/Suriname using only IMS was 0.4695; when using AMS, this was slightly higher (0.5092). The differences in Mantel's r coefficient were smaller for Ecuador (0.4029 and 0.4039) and French Guiana (0.7944 and 0.7987).

**Figure 3:**
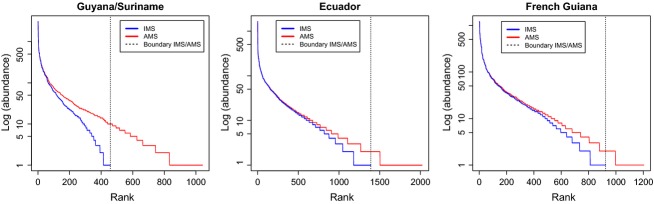
Rank abundance curves for the IMS (blue) and AMS dataset (red) for Guyana/Suriname (upper left), Ecuador (upper right), and French Guiana (bottom left), showing the effect of omitting UMS. The AMS dataset contains many more rare species and the UMS are mostly in the tail of the distribution as indicated by the dashed line separating the truncated IMS datasets and the AMS datasets, effectively transforming the curve from a logseries to a lognormal.

### Using higher-taxon-level resolution in comparison with identified morpho-species

**Figure 4:**
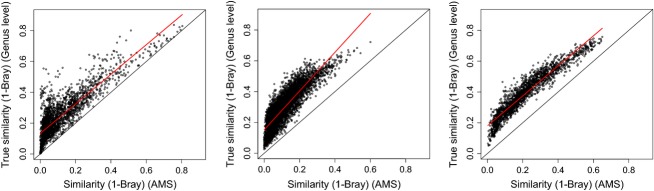
Comparisons between pairwise similarities between all plot pair combinations using a higher-taxon-level indicator (here genus level) and the AMS dataset (Guyana/Suriname topright, Ecuador bottom left, and French Guiana bottom right). Although patterns still remain the same, similarities are continuously higher than expected based on AMS when using only higher taxa as an indicator. Results show that using only IMS in comparison with AMS gives a better fit.

Using higher-taxon-level (genus-level) data, similarities among communities are higher and much more deviant from the expected similarities based on AMS (Fig. 4) than with the IMS (Fig. 2). The latter shows a very strong linear regression, while regressions between similarities based on genus level appear to predict the pattern generated by AMS not as good (with *R*^2^ values ranging from 0.74–0.90, Table 2) as using only the IMS.

### Predictions of Multivariate analyses

Nonmetric multidimensional scaling of all three subsets showed good segregation along the first two axes of the NMDS when using AMS as well as when using only IMS. Axis 1 scores derived from only the IMS, and AMS were very similar (Fig. 2). All linear regressions of first axis scores for the AMS and IMS NMDS yielded adjusted *R*^2^ values of >0.97, for all three datasets. The same pattern emerged from using the second axis with (*R*^2^ values of >0.99) (Table 2). In all cases except French Guiana, deviation of the slopes from 1 was found not to be significant using a 95% confidence interval. Although for French Guiana, the CI was between 0.984–0.993. Examples of NMDS results for all three datasets using either AMS or IMS can be found in the SOM.

### Robustness of predictions: data stratification

IMS made up between 44–77% of all species encountered in the datasets (above). After randomly selecting 50% and 25% of all IMS from each dataset and recalculating the distance decay in similarity and Mantel's statistic using the Bray–Curtis, Sørensen, and Jaccard index, regressions dropped slightly, but they still yielded high linear regression coefficients (Table 2). For Guyana/Suriname, 50 runs with 50% of IMS yielded adjusted *R*^2^ values between 0.60 and 0.80 for the tree indices. Ecuador and French Guiana yielded even higher *R*^2^ values for each index, ranging from 0.85–0.92. In the case of 25% of IMS drawn randomly from the total set of IMS, this gave a mean linear regression coefficient *R*^2^ between 0.51 and 0.59 for Guyana and in the ranges 0.75–0.79 and 0.71–0.82 for Ecuador and French Guiana, respectively.

## Discussion

We asked if omitting individuals that have no valid species name (UMS) from ecological datasets would change the overall result of several important ecological analyses. We showed that when using only the IMS of actual field data, major ecological patterns such as the differences in species richness among sites, floristic similarities among sites, and ordination gradients in species composition were maintained. The linear regressions between analyses based on the IMS only or AMS (including all UMS) were extremely high for almost all analyses (*R*^2^ > 0.91). This was the case even when simulating a larger fraction of UMS. And although FA underestimated species diversity, when using only IMS, linear regressions between FA from IMS and AMS still showed extremely high *R*^2^ values, suggesting that spatial patterns in diversity will still be similar when using only IMS. However, if individuals can be assigned to morpho-species within plots, this will also allow the comparison among plots from different resources (ter Steege et al., 2003), including the UMS.

Different methods have been proposed in the past to deal with unidentified morpho-species. By relaxing the taxonomic resolution (Terlizzi et al. 2003), however, the prediction of similarity between our sites was lower than when omitting UMS (Figs 2 and 4). Thus, although a genus-level approach allows a larger number of individuals from the dataset to be used, its performance was not necessarily better. Cayuela et al. (2011) used a different method of trimming UMS from a dataset: instead of omitting individuals of UMS, they randomly reassigned them to species present in other plots (or to itself again, in which case it was considered a new species). This resulted in plots becoming more similar then observed as all plots drew the names for the indets from a panmictic species pool. Omitting UMS results in lower similarities, rather than higher but with smaller deviation (cf. Fig 1 Cayuela et al. 2011).

When UMS are omitted, a risk is introduced of underestimating the actual geographic range of the species, for example, when these UMS are located at the range margins. It would then be expected that this would greatly influence the agreement in similarity of species composition between IMS only and AMS (Pitman et al. 1999). However, this effect appears to be negligible in terms of determining patterns of tree species turnover, as shown by our extremely high regression coefficients between similarities among plots based on AMS and IMS alone (Fig. 2). For the sake of argument, there is a slight decrease in the correlation (Mantel r) if only IMS are taken into account in the analysis, but this effect arguably does not change the patterns of species turnover. Confidence intervals for the slope of the regression for the comparison of similarity values based on all three used indices showed that with an increasing amount of species identified (i.e., a lower proportion of UMS) as is the case with subsequent increased IMS when comparing the Guyana/Suriname, Ecuador, and French Guiana datasets, the linear regression starts to approach a slope of 1. As example, with 77% identification of all species in French Guiana, a confidence interval of 0.979–0.982 shows that the slope of the regression between IMS and AMS similarity values calculated with the Bray–Curtis index is extremely close to a slope of 1, indicating that the Bray–Curtis similarity values are nearly equal between the IMS and AMS dataset. This was also true when using the other indices.

The similarity matrices are the input for the distance decay in similarity, Mantel test, and NMDS. As a result, it is obvious to expect that if the similarity matrices are very similar, these will also generate very similar results when AMS and IMS are compared. We, however, did not know this a priori and had decided to show all three analyses as primary examples because they are all often used by ecologists. For almost all analyses (except NMDS first axis comparison for Guyana and Ecuador), there was a significant positive deviation from the relation *y* = *x* with slope 1, when comparing results of AMS and IMS. Hence, omitting species has a small but significant effect. However, this difference is apparently not enough to distort the actual pattern of species turnover. Results from the Raup–Crick analyses also showed that using both approaches to calculate the distance matrices, that is, with and without permutation based on frequency-dependent probabilities of selecting species to be used for Mantel's r, still yields similar results. There are some limitations to using this method, however. As it is a presence/absence-based nonmetric measure, identical samples can have dissimilarities above zero and samples sharing no species can have dissimilarities less than one. Samples sharing rare species in particular appear to be more similar as the probability of sharing these species is lower in comparison with samples sharing more common species and data are always treated as presence/absence. In addition, Lennon et al. (2001) showed that strong local differences (i.e., in adjacent plots) in species richness might have an influence on species similarities when using the Sørensen index (Lennon et al. 2001). But even in light of these limitations, the results from the similarity analyses indicate that, while leaving out unidentified species might compromise species ranges, it does not seem to affect overall similarity, thus remaining a useful approximation for similarity analyses. Results from the NMDS indeed supported the other analyses. Scores from the first axis of the NMDS were nearly identical between only the IMS and AMS. This was also true for the second axis scores. As regressions between NMDS scores of both the first and second axes showed extreme good regression coefficients (*R*^2^ values all >0.97), it shows that it is in fact possible to omit UMS from datasets without losing large-scale patterns as are analyzed when using NMDS. If a strong underlying gradient, for instance, due to different forest types, would be responsible for the robustness of patterns, they could be maintained if a large enough fraction of plots in each forest type is still present after omitting UMS. Table 1, however, shows a summary of the datasets used and the types of forest incorporated in the analyses, and although five different types of forest Igapó (IG), Podzol (PZ), Swamp (SW), Terra Firme (TF), and Várzea (VA) were used, the far majority of plots is on Terra Firme soils suggesting forest types are not likely the reason for maintaining these patterns.

## Common Species Dominate Ecological Patterns

Even when simulating a larger proportion of the complete dataset to be unknown, the majority of analyses still yielded very comparable results. Considering this simulated loss of information, this suggests that patterns of species diversity and composition are robust enough to emerge from (very) limited datasets. Most likely, this is due to the fact that common species are common enough to even have a pattern, whereas rare species are often so restricted that they do not affect the large-scale patterns much. Lennon et al. (2001) already showed that the more common species were mostly responsible for richness patterns in avian species (Lennon et al. 2001). It would appear that in tropical tree species, the common species also dominate major ecological patterns such as species turnover. Even when using the Jaccard index for similarity, which is only based on the presence or absence, results from the similarity analyses showed that omitting UMS made no difference in the overall result (although deviation from the relationship *y* = *x* was significant). If IMS consist mostly of common species, this common species domination as explained above would explain why using only IMS results in the same patterns as when using AMS. To test this, we plotted a rank abundance curve on a logarithmic scale. It becomes immediately apparent (Fig. 3) that the AMS dataset include many more rare species than did the IMS subset. In fact, omitting the UMS from the dataset results in the rank abundance curve showing a lognormal distribution instead of the logseries distribution when AMS are plotted. In a sense, omitting UMS truncates the datasets from the right, cutting of the rare species. This also explains why our results for Fisher's alpha showed an underestimation when using only IMS and why similarities between plots using just IMS and AMS deviate with increasing similarity. UMS are not randomly distributed among the common and rare species but are mostly rare species. Hence, FA calculated with N and S for just the IMS will generally be an underestimate.

Finding near identical similarities of species composition and patterns from NMDS results suggest that patterns of similarity and thus composition are robust. Although Fisher's alpha based on IMS or AMS showed nearly identical spatial patterns, using a dataset with AMS is still preferred, as FA is not based on comparison and will be underestimated when using only IMS. Overall, the results presented here suggest that irrespective of metrics used, analyses and their limitations; strong ecological patterns still arise using only IMS. In other words, having a large number of unidentified species in a dataset may not affect our conclusions as much as is often thought. However, this should not be interpreted as an argument to omit all UMS all the time. They remain important as they may represent important species (Mouillot et al. 2013) and are essential for the calculation of correct diversity measures.

## Supporting Information

**Figure S1.** Example showing the distance decay in similarity (DDS) for the Guyana/Suriname dataset based on the distance matrices calculated with the Bray-Curtis index used for the Mantel statistic Analysis of DDS are shown for only IMS (upperleft), AMS (upperright) and the linear regression for Guyana/Suriname (lowerleft). **Figure S2.** Example showing the distance decay in similarity (DDS) for the Guyana/Suriname dataset using the Raup-Crick analyses. Analysis of DDS are shown for only IMS (upperleft), AMS (upperright) and the linear regression for Guyana/Suriname (lowerleft). **Figure S3.** Example showing the distance decay in similarity (DDS) for the Ecuador dataset based on the distance matrices calculated with the Bray-Curtis index used for the Mantel statistic Analysis of DDS are shown for only IMS (upperleft), AMS (upperright) and the linear regression for Ecuador (lowerleft). **Figure S4.** Example showing the distance decay in similarity (DDS) for the Ecuador dataset using the Raup-Crick analyses Analysis of DDS are shown for only IMS (upperleft), AMS (upperright) and the linear regression for Ecuador (lowerleft) **Figure S5.** Example showing the distance decay in similarity (DDS) for the French Guiana dataset based on the distance matrices calculated with the Bray-Curtis index used for the Mantel statistic Analysis of DDS are shown for only IMS (upperleft), AMS (upperright) and the linear regression for French Guiana (lowerleft). **Figure S6.** Example showing the distance decay in similarity (DDS) for the French Guiana dataset using the Raup-Crick analyses Analysis of DDS are shown for only IMS (upperleft), AMS (upperright) and the linear regression for French Guiana (lowerleft).
