# Supplementary material for: Are all species necessary to reveal ecologically important patterns?
Source: Ecol Evol. 2014 Dec 2;4(24):4626–36. doi: 10.1002/ece3.1246 (PMC4278815; doi:10.1002/ece3.1246)
Supplement: Supplementary file 1 [file ece30004-4626-sd1.docx]

*Supplementary Online Material (SOM):*

*Figure S1:*

Example showing the distance decay in similarity (DDS) for the Guyana/Suriname dataset based on the distance matrices calculated with the Bray-Curtis index used for the Mantel statistic. Analysis of DDS are shown for only IMS (upperleft), AMS (upperright) and the linear regression for Guyana/Suriname (lowerleft)

*Figure S2:*

Example showing the distance decay in similarity (DDS) for the Guyana/Suriname dataset using the Raup-Crick analyses. Analysis of DDS are shown for only IMS (upperleft), AMS (upperright) and the linear regression for Guyana/Suriname (lowerleft)

*Figure S3:*

Example showing the distance decay in similarity (DDS) for the Ecuador dataset based on the distance matrices calculated with the Bray-Curtis index used for the Mantel statistic. Analysis of DDS are shown for only IMS (upperleft), AMS (upperright) and the linear regression for Ecuador (lowerleft)


*Figure S4:*

Example showing the distance decay in similarity (DDS) for the Ecuador dataset using the Raup-Crick analyses. Analysis of DDS are shown for only IMS (upperleft), AMS (upperright) and the linear regression for Ecuador (lowerleft)


*Figure S5:*

Example showing the distance decay in similarity (DDS) for the French Guiana dataset based on the distance matrices calculated with the Bray-Curtis index used for the Mantel statistic. Analysis of DDS are shown for only IMS (upperleft), AMS (upperright) and the linear regression for French Guiana (lowerleft)

*Figure S6:*

Example showing the distance decay in similarity (DDS) for the French Guiana dataset using the Raup-Crick analyses. Analysis of DDS are shown for only IMS (upperleft), AMS (upperright) and the linear regression for French Guiana (lowerleft)

*Figure S7:*

Example showing the Non Metric Muldimensional Scaling (NMDS) ordination procedure for Guyana/Suriname (upper), Ecuador (middle) and French Guiana (lower) using meta-MDS. Analyses are shown for only IMS (left) and AMS (right). Dashed lines indicate different grouping based on country (Guyana/Suriname), forest type or geographic subdivision (North/South).
